# Book Reviews

**Published:** 1904-10

**Authors:** 


### BOOK REVIEWS.

BRAVEST OF THE BRAVE—CAPTAIN CHARLES DE LANGLADE, by Publius P V. Lawson, published by Log Cabin Inn, Menasha, Wis.; two hundred and ninety-eight pages, seventeen full page colored illustrations, cloth $1.50, morocco $3.00.

This is not fiction but history lived in the great lake region that reads like romance. Its scenes are laid in the stirring days of the habitant and trader; the long war for the conquest of Canada; thrilling days in the contest for the fur trade; where the bushranger, the savage, and war, massacre, and the beaver formed the current topic of interest; and Pontiac with firebrands and tomahawk swept the frontier; then the echoes of the Revolution stir the west, and the savage, who hovers over all Colonial history, hurries to the massacre of St. Louis. The scenes shift over Canada, the great lakes, the Ohio and Mississippi Valley, now Wisconsin, Michigan, Illinois, Missouri, etc. A remarkably interesting and instructive story, well told.

FOOD IN HEALTH AND DISEASE.
by I. Burney Yeo, M. D., F. R. C. P.

Emeritus Professor of Medicine in King's College, London, and Consulting Physician to King's College Hospital, Etc., Etc. Ninth Edition, with Illustrations. Chicago, W. T. Keener & Co. $2.50 net.

The book is divided into two parts, the first devoted to Food in Health, the second to Food in Disease. In the first part are included the important subjects of Army and Prison Dietaries, School Dietaries and Feeding during the critical period of Infancy and Childhood. In the second part are included Feeding in Acute Disease and Convalescence, Dietetic Treatment of Obesity, Anaemia, Neurasthenia, Hospital Dietaries, etc. It is a good book, worthy of careful study.

